# Complete Genome Sequence of Genotype Psittacine Beak and Feather Disease Virus, a Strain Identified from Budgerigars in China

**DOI:** 10.1128/MRA.00040-19

**Published:** 2019-05-16

**Authors:** Rujia Cheng, Yaqing Mao, Qiuchen Li, Shijie Xie, Yingju Xia, Haojie Sun, Kai Niu, Shijing Sun, Jie Li, Yu Feng, Xiaowei Peng, Hui Jiang, Liangquan Zhu, Xuezheng Fan, Zhanye Lin, Jiabo Ding, Yuming Qin, Guanlong Xu

**Affiliations:** a National Reference Laboratory for Animal Brucellosis, China Institute of Veterinary Drug Control, Beijing, China; b College of Veterinary Medicine, Shandong Agricultural University, Tai’an, Shandong, China; c Department of Population Medicine and Diagnostic Sciences, College of Veterinary Medicine, Cornell University, Ithaca, New York, USA; d Animal Husbandry and Veterinary Bureau, Ministry of Agriculture and Rural Affairs, Beijing, China; KU Leuven

## Abstract

Psittacine beak and feather disease virus (PBFDV) has been reported in many countries, such as Australia, Poland, the United States, South Africa, etc. In this study, the complete genome of a PBFDV isolate was determined and characterized from budgerigars in China.

## ANNOUNCEMENT

Psittacine beak and feather disease virus (PBFDV) is a nonenveloped virus with a diameter of 14 to 16 nm and belongs to the genus *Circovirus* in the family *Circoviridae*. The genome of PBFDV is a 2-kb circular single-stranded DNA molecule possessing two major open reading frames (ORFs) encoding a replication-associated protein (Rep) and the capsid protein (Cap) (1, 2). Psittacine beak and feather disease (PBFD) is characterized by immune suppression, loss of feathers, weight loss, and the development of morphologically abnormal feathers, and it sometimes leads to sudden death in young birds (3, 4).

PBFDV is distributed worldwide with a wide range of hosts, including more than 60 species of psittacine birds (5). PBFDV has been reported in many countries, such as Australia, Poland, the United States, South Africa, etc. (6–8). In this study, the full-genome sequence of PBFDV isolated from budgerigars in Shandong Province, China, was determined and analyzed. Clinical signs of feather loss were observed in budgerigars at a breeding facility. The feather samples were collected and homogenized, and total DNA was extracted from the feather samples using a QIAamp minikit (Qiagen, Hilden, Germany). A primer pair (forward, 1,368 bp-TTGGGTCCTCCTCCTATCGGGATC-1,392 bp; reverse, 1,868 bp-AGACACCGTTTTACAACCAATAG-1,843 bp) targeting 500 bp of the capsid gene was designed using Primer Premier version 5.0 (Canada). The DNA samples were screened using PCR, and the PCR products were sequenced using Sanger sequencing by the TsingKe Biological Technology Company (Beijing, China). All of the clinical samples were positive for the identical sequence; this indicated that these birds were infected with PBFDV. To further characterize the pathogen, the full genome of one psittacine beak and feather disease virus isolate in the positive samples (named PBFDV-GD) was sequenced and analyzed. DNA isolated from the samples was directly amplified using a second pair of primers to cover the full genome (forward, 1 bp-GTTATGTAGTCAGAATTCCAAATTA-25 bp; reverse, 2,000 bp-GTTAGCTAAAATGGAAATGAGGCCCAC-1,973 bp). The resulting PCR product (2 kb) was sequenced using Sanger sequencing in both directions and walking primers. The final genome was assembled and analyzed with the Lasergene software version 7.0 (DNAStar, USA) (9).

The genome of the PBFDV-GD isolate was found to be 2,000 bp long, with 52% GC content, and contains two ORFs which encode Rep (289 amino acids [aa]) and Cap (237 aa). Our data showed that PBFDV-GD shares about 77% to 90% similarity with the PBFDV genomes deposited in GenBank from Australia, South Africa, and New Zealand, suggesting that the PBFDV circulating in China is different from those PBFDV strains found in Australia, South Africa, and New Zealand.

Given the extensive international bird trade, PBFDV has spread worldwide and poses a threat to psittacine birds. The PBFDV strain identified in the diseased budgerigars indicates that a different lineage of PBFDV is circulating in China. Although PBFDV was identified in the diseased budgerigars, it remains to be confirmed whether PBFDV was the sole cause of the observed disease. Further active surveillance is needed to determine the prevalence of PBFDV in China.

### Data availability.

The complete genome sequence reported here was submitted to GenBank under the accession number MK120438.
